# Task Sharing and Shifting to Provide Pathology Diagnostic Services: The Kenya Fine-Needle Aspiration Biopsy Cytology and Bone Marrow Aspiration and Trephine Biopsy Training Program

**DOI:** 10.1200/JGO.18.00094

**Published:** 2018-11-06

**Authors:** Shahin Sayed, Andrew Field, Jamilla Rajab, Anderson Mutuiri, Jessie Githanga, Mary Mungania, Nancy Okinda, Zahir Moloo, Abubakar Abdillah, Brian Ayara, Erick Chesori, Julia Muthua, Leah Obosy, Thaddeus Massawa, Okoth Obiero, Elizabeth Kagotho, Peter K. Shikuku, Andrew K. Gachii, Eunida Migide, Donstefano Muninzwa, Sanford M. Dawsey, Lucy Muchiri

**Affiliations:** **Shahin Sayed**, **Anderson Mutuiri**, **Nancy Okinda**, **Zahir Moloo**, **Abubakar Abdillah**, **Erick Chesori**, **Elizabeth Kagotho**, **Eunida Migide**, and **Donstefano Muninzwa**, Aga Khan University Hospital; **Jamilla Rajab**, **Jessie Githanga**, **Mary Mungania**, **Brian Ayara**, **Julia Muthua**, **Leah Obosy**, **Thaddeus Massawa**, **Peter K. Shikuku**, and **Lucy Muchiri**, University of Nairobi; **Okoth Obiero** and **Andrew K. Gachii**, Kenyatta National Hospital, Nairobi, Kenya; **Andrew Field**, Notre Dame University Medical School and St. Vincent’s Hospital, Sydney, Australia; and **Sanford M. Dawsey**, National Cancer Institute, Bethesda, MD.

## Abstract

**Purpose:**

Fine-needle aspiration biopsy (FNAB) cytology is a simple, inexpensive, and accurate diagnostic test for benign, infectious, and malignant lesions of the breast, thyroid, lymph nodes, and other organs. Similarly, bone marrow aspiration and trephine (BMAT) biopsy procedures are relatively simple and inexpensive techniques that are important for diagnosing and monitoring many hematologic diseases including leukemias and lymphomas. However, the scarcity of pathologists in Kenya limits patient access to these simple diagnostic tests. We describe a task sharing and shifting program that sought to improve the provision of FNABs and BMAT biopsies in tertiary public hospitals in Kenya.

**Methods:**

Between January 2016 and February 2017, we trained pathologists, pathology residents, and technologists from the University of Nairobi and Aga Khan University Hospital, Nairobi, in FNAB and BMAT biopsies, who in turn trained pathologists, medical officers (MO), clinical officers (CO), and technologists at five tertiary public hospitals. The program involved curriculum development, training workshops, the establishment of new and strengthening existing FNAB and BMAT biopsy clinics, interim site visits, audits, and stakeholder workshops.

**Results:**

Fifty-one medical personnel at the tertiary hospitals were trained. The FNAB numbers increased by 41% to 1,681, with 139 malignant diagnoses (7.1%). BMAT biopsy numbers increased by 268% to 140, with 34 malignant cases. Between 60% and 100% of the FNAB and BMAT biopsy procedures were performed by MO and CO over the project period. One new FNAB and two new BMAT biopsy clinics were established.

**Conclusion:**

This project demonstrates a successful model of task sharing and shifting from specialist pathologists to MO and CO that improved access to important FNAB and BMAT biopsy services in a low-resource setting.

## INTRODUCTION

Similar to other developing^1,2^ countries, the burden of cancer in Kenya is on the rise, with an estimated 37,000 new cancer cases and 28,500 cancer deaths in 2012.^1^ Cancer-related mortality is now the third-leading cause of death after infections and cardiovascular diseases.^3^ The key to appropriate cancer treatment is accurate diagnosis.^4^ However, there are fewer than one pathologist per 500,000 people, compared with one pathologist per 15,000 to 20,000 people in the United States and the United Kingdom,^5^ and one report estimates that it would take more than 400 years to increase the pathologists-to-population ratio in the region to that of the United States or the United Kingdom.^6^

Traditionally, in Kenya, simple diagnostic procedures for cancer, such as fine-needle aspiration biopsy (FNAB) cytology and bone marrow aspiration and trephine (BMAT) biopsy, have been performed almost exclusively by pathologists at county hospitals, which are tertiary health facilities. Broader access to FNABs and BMAT biopsies is thus limited, largely because there are few well-trained personnel to perform these diagnostic techniques.^7^

Task sharing and shifting through training nonpathologist medical and paramedical staff to perform procedures such as FNAB and BMAT biopsy may overcome some of the challenges associated with the scarcity of pathologists in low- and middle-income countries (LMICs).^8^ Task sharing and shifting has been demonstrated previously to be useful for training laboratory technologists to process tumor specimens^9^ and in the delivery of HIV care.^10^

We describe a task sharing and shifting program for FNAB and BMAT biopsy procedures that was developed by the pathology subtrack members at a National Cancer Stakeholders’ meeting held in 2014 in Naivasha, Kenya, jointly supported by Kenya’s Ministry of Health and the US National Cancer Institute.^11^ We evaluated the effects of this program by the change in the number of skilled personnel performing FNAB and BMAT biopsy procedures, the number of FNAB and BMAT biopsy procedures performed, the rate of unsatisfactory FNAB and BMAT biopsy samples, the diagnosis turnaround time (TAT), and the effect on the capacity for quality cytology processing at these facilities.

## METHODS

### Program Overview

**Fig 1:**
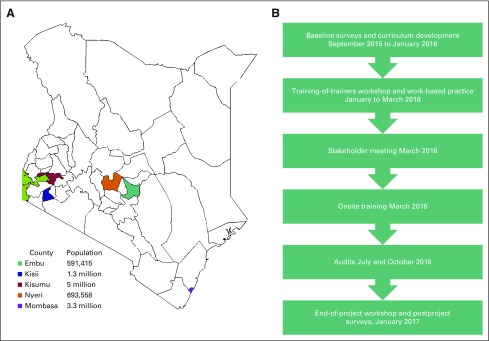
(A) Counties participating in the fine-needle aspiration biopsy (FNAB) cytology and bone marrow aspiration and trephine (BMAT) biopsy training program. (B) Overview of the FNAB and BMAT biopsy training program.

This was a partnership between Aga Khan University Hospital, Nairobi (AKUHN) and the University of Nairobi (UoN), supported by collaborators from the Center for Global Health at the US National Cancer Institute and St. Vincent’s Hospital and Notre Dame University Medical School, Sydney, Australia. The program was implemented over a 14-month period from January 2016 through February 2017 and initially included four participating county health facilities: Coast Provincial General Hospital (CPGH) in Mombasa, Nyeri Provincial General Hospital (NPGH) in Nyeri, Jaramogi Oginga Odinga Teaching and Referral Hospital in Kisumu, and Embu Provincial General Hospital (EPGH) in Embu. A fifth site, the Kisii Teaching and Referral Hospital (KTRH) in Kisii, was added in July 2016 (Fig 1).

The overall aim of the program was to provide diagnostic support for cancer care, using a two-step approach: first, a training-of-trainers workshop trained pathology residents from the UoN and AKUHN and practicing pathologists from the five participating hospitals to become trainers in FNAB and BMAT biopsy techniques; and second, these new trainers trained additional pathologists, medical officers (MO), and clinical officers (CO) in the five county hospitals to perform quality FNAB and BMAT biopsy procedures. MO are first- and second-year postinternship doctors who work in county hospitals for a mandatory 3 years after their medical graduation. CO are career paramedical officers who work in county hospitals after completing a 3-year diploma or degree.

In parallel, laboratory technologists were trained in the handling and processing of FNAB and BMAT biopsy samples by experienced laboratory technologists from UoN and AKUHN. The program overview is outlined in Figure 1B.

### Development of Survey Tools and Curricula

#### Survey tools.

The project team developed pre- and post–FNAB and BMAT biopsy training survey tools that assessed the status of FNAB and BMAT biopsy services in participating facilities between September and November 2015.

#### Training curricula.

Training curricula on FNAB and BMAT biopsy procedures were developed through online consultations by the project team over 3 months beginning in September 2015. The curriculum included the theory and technical aspects of FNAB and BMAT biopsy procedures, indications for the procedures, and quality assurance requirements.

### Program Implementation

#### Training-of-trainers workshop.

A 3-day training workshop was conducted in January 2016 at UoN to train the pathology residents and county pathologist trainers. The training started with a precourse multiple-choice test and a practical evaluation of the trainees’ ability to perform FNAB and BMAT biopsy procedures, followed by hands-on workbench and clinical training in FNAB and BMAT biopsy techniques, with trainees performing one to two FNABs in an organized FNAB clinic under the direct supervision of an international instructor (AF). Each participant also performed one to two BMAT biopsy procedures on prebooked patients under the supervision of faculty. A parallel course in the preanalytic and analytic techniques needed for quality FNAB and BMAT biopsy specimen processing was conducted for senior technologists from both the UoN and AKUHN. Then there was a teach-back session to assess the teaching ability of the workshop participants, which was followed by administration of a confidence rating tool that was based on the Likert scale (0 is not confident and 5 is very confident) to evaluate the confidence levels of the newly trained trainers. Postcourse evaluation of the participants (practicing and resident pathologists) was conducted at the end of the 3-day training through practical assessment of competency in the FNAB technique. In addition, the trained residents each completed at least 10 BMAT biopsy and 20 FNAB procedures between January and March 2016 at Kenyatta National Hospital under the supervision of faculty, which was followed by a final work-based competency assessment.

#### On-site training at county hospitals.

Pathology resident trainers and on-site county pathologists used the same curriculum and 3-day training program to train interested MO and some CO attached to the participating health facilities. The trainers also undertook a follow-up visit 6 weeks later to provide supervision and, where necessary, revision of parts of the training.

### Program Evaluation

The program was evaluated through pre- and post-training surveys and mid- and end-of-project audits and quality-assurance challenges (Fig 1B). Quality indicators included the number of FNAB and BMAT biopsy procedures performed over the project period, the rate of unsatisfactory FNAB and BMAT biopsy samples, the diagnosis TAT, and the number of malignancies diagnosed. However, there was no formal surgical follow-up of FNAB malignant diagnoses because this was beyond the remit of this study.

#### Administration of survey tools.

The pathology residents administered the previously developed baseline and postproject surveys to MO, CO, pathologists, technologists, and hospital leadership at all four original participating sites. The surveys examined the impact of the program on the provision of FNAB and BMAT biopsy service at these facilities.

#### Audits.

Two audits were conducted, a midterm audit at all sites and an end-of-project audit at CPGH and EPGH. Fewer facilities were included in the end-of-project audit because of time constraints. The auditors (AF, PKS) used FNAB and BMAT biopsy audit checklists developed by the audit teams and the project leads. Each audit visit to a county hospital incorporated the following: a preaudit morning meeting with the trainees and medical administrator, the auditors’ practical structured assessment of each MO, remedial instruction as required, work-based assessment by the auditors of the MO as they performed FNAB and BMAT biopsy procedures in clinics, and a feedback and discussion meeting at the end of each audit day

#### Technical staff external quality assurance.

Two technical challenges, consisting of unstained direct FNAB smears and formalin-fixed paraffin-embedded trephine biopsies, were sent to participating sites to assess the technical staff on proficiency in sample handling and processing.

### Program Dissemination

#### Initial stakeholders’ meeting.

A stakeholders’ meeting of the county health directors, medical superintendents, and pathologists from all four original participating sites was held on March 1, 2016, at AKUHN to obtain a commitment for the project. The agenda included introducing the project to the stakeholders, presenting the expected benefits and the roles and responsibilities of the participating teams, and proposing an income-generation model that the county hospitals could adopt.

#### End-of-project workshop.

A stakeholder and participant workshop was held in January 2017 to review the performance of participating sites, the achievements of the project, the shared challenges, the lessons learned, and the proposed changes for an improved model for the project going forward.

## RESULTS

### Training

A total of 23 participants, including nine pathologists, six pathology residents, and eight technologists, were trained as trainers of trainers in the FNAB and BMAT biopsy techniques. All 23 trainers showed improvement between their initial and post-training assessments of FNAB and BMAT biopsy technique (Table 1). The trainers, in turn, trained a total of 51 medical personnel, including two pathologists, 33 MO, two CO, and 14 technologists in participating county hospitals.

**Table 1:**
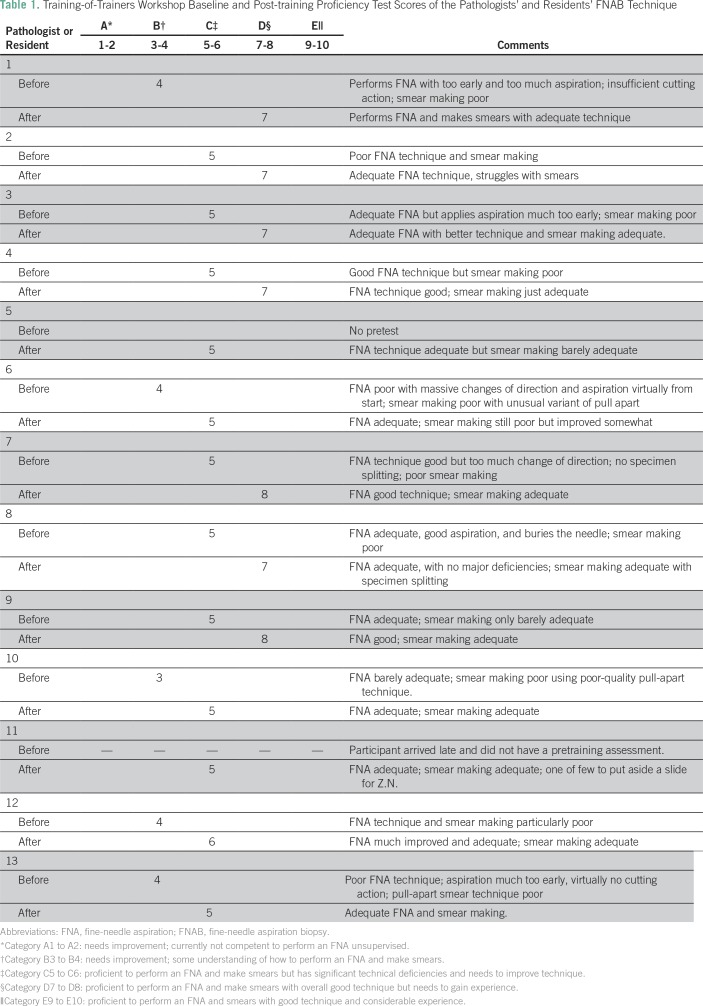
Training-of-Trainers Workshop Baseline and Post-training Proficiency Test Scores of the Pathologists’ and Residents’ FNAB Technique

### FNAB and BMAT Biopsy Procedures Performed

The number of FNABs performed between March/April and November 2016 showed an overall increase of 41% across all facilities compared with the same period in 2015 (Table 2). In addition, the FNAB service at KTRH was established only after the training of their MO, who performed 181 FNABs over the project period.

**Table 2:**
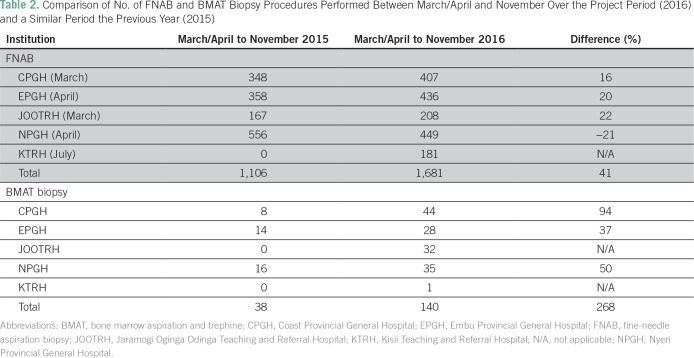
Comparison of No. of FNAB and BMAT Biopsy Procedures Performed Between March/April and November Over the Project Period (2016) and a Similar Period the Previous Year (2015)

There was an even more marked increase in the number of BMAT biopsies at the training sites, from a total of 38 procedures between March/April and November 2015 to 140 procedures over the same period in 2016, an increase of 268% (Table 2). Furthermore, trephine biopsies were performed for the first time in two of the facilities.

Overall, the rate of unsatisfactory FNABs decreased from an average of 14% before on-site training to 8% soon after the training and to 4% several months later after the auditing process and retraining (Table 3). These unsatisfactory rates compared favorably with the rates at UoN and AKUHN and in the general FNAB literature. However, the rates of unsatisfactory FNABs were still high at the end of the project at NPGH (16%), where there was no supervising pathologist, and at KTRH (19%), which joined the program later and did not benefit from the audit or retraining. Of the total number of 140 BMAT biopsies performed, only 18 (14%) were unsatisfactory for evaluation.

**Table 3:**
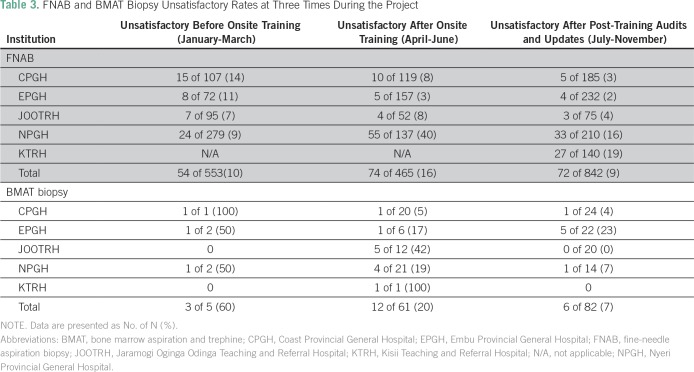
FNAB and BMAT Biopsy Unsatisfactory Rates at Three Times During the Project

### Diagnostic Categories of FNAB and BMAT Biopsies in 2016

Table 4 presents the diagnostic categories for each procedure by participating site. The proportion of malignant lesions in all FNABs ranged from 4% to 12%, depending on the mix of patients and the range of clinics where FNAB was performed at each participating center (Table 4). A total of 34 malignancies (24%) were diagnosed on BMAT biopsy samples.

**Table 4:**
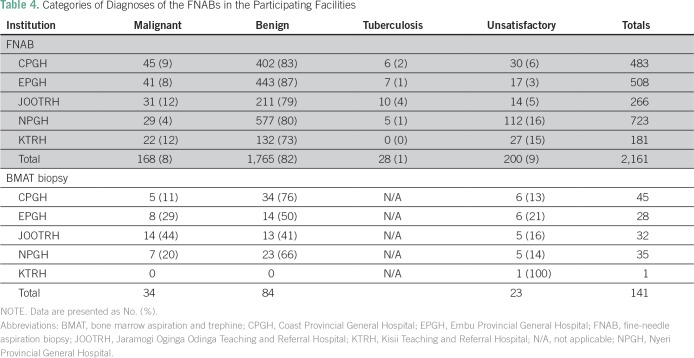
Categories of Diagnoses of the FNABs in the Participating Facilities

### Average TATs

The TAT for FNAB in the project period was compared with a similar period the previous year (Table 5). The TAT for FNAB at CPGH (10 days) and EPGH (5 days) did not change. At NPGH, the TAT increased to an average of 19 days, because the pathologist had left and the slides had to be sent to Nairobi for reporting. The TAT for bone marrow aspirates decreased significantly across all sites, by an average of 50% (from 7 to 3 days), with the exception of NPGH, where all of the samples were sent to Nairobi for reporting. It took longer across all sites for trephine biopsy reports, with an average TAT of 2 to 3 weeks.

**Table 5:**
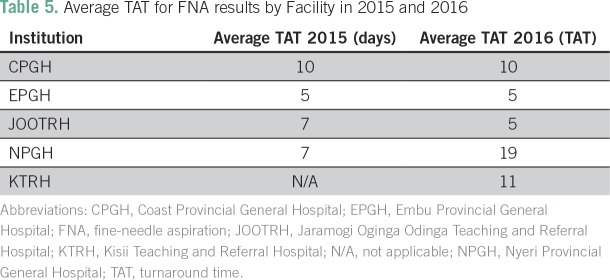
Average TAT for FNA results by Facility in 2015 and 2016

### Baseline and Postproject FNAB and BMAT Biopsy Surveys

At baseline, all the FNABs were performed by a pathologist, and by the end of the project, the 33 MO and two CO were performing 60% to 100% of the FNAB and BMAT biopsy procedures. The FNAB smear-making technique was described before training as a squash and smear technique, and this was replaced by the far superior split sample specimen procedure taught by the international instructor (AF).

## DISCUSSION

Our program provides an example of how task sharing and shifting can address some of the challenges associated with the shortage of pathologists in LMIC. Task sharing and shifting is a well-established strategy in other areas of health care,^10,12^ and a study from Rwanda demonstrated how task sharing and shifting of technical skills in anatomic pathology laboratories can influence TAT through the efficient use of available staff.^13^

Involving pathology residents as trainers was a strength of this project and had several benefits. The residents reinforced and improved their procedural skills and became empowered as trainers, while also learning project implementation and report writing. For county hospital pathologists, the project reinforced their procedural skills and potentially improved their reporting ability in FNAB and BMAT biopsy. Importantly, one site (NPGH), where the pathologist left, was able to continue providing FNAB and BMAT biopsy services using a project-trained MO and two CO whom he had trained to procure samples, which were then evaluated at a referral laboratory.

Where there were deficiencies noted during the interval audits, remedial training was provided. For BMAT biopsies, the lack of adequate numbers of patients throughout the training period meant that experiential improvement was still a challenge. Furthermore, to have an impact on TAT, local pathologists will require additional training in reporting BMAT biopsy specimens.

Quality assurance was a major component of this project. The audits conducted by the external team, the two external quality-assurance challenges for technical staff, and the feedback sessions were integral to ensuring that the quality chain from the time of sample requisition to the time of result availability was maintained for appropriate and timely patient management.

In all the county hospitals, the training of MO and CO in these procedures added to their workload and challenged their routine work rosters. Initially, no allowances were made by the medical administration for MO to perform these procedures. Cover had to be provided by their fellow MO, and this situation continued throughout the program, but the motivated MO found time to service the FNAB clinics effectively. During audit meetings, these matters were discussed to a degree, and at the final stakeholders’ meeting there was apparent recognition by the medical administrators of the increased workload.

Some pathologists also viewed the enhanced FNAB and BMAT biopsy service as an increase in their workload and, particularly with BMAT biopsies, a challenge to report. In these referral hospitals, the pathologists provide forensic autopsy services that require frequent court attendances, which poses a challenge for increasing any of their other duties. Similarly, some medical administrators perceived increasing diagnostic tests as an increase in costs, although adult patients were charged an equivalent of 5 to 7 USD for FNAB and BMAT biopsy services, which should have covered the costs. Hospital administrators should be encouraged to reinvest income from billing for FNAB and BMAT biopsy procedures into their pathology laboratories for purchase of consumables that are needed to sustain the service. The study demonstrated that there is a need during program initiation for administrators to fully understand the benefits and local requirements of the program to encourage buy in.

In the county hospitals, the CO are the most stable cadre of staff, and training only a few from the outset limited the impact of the project, especially when MO transferred out or left for postgraduate training or were on annual leave. The situation was compounded in late 2016 by a nationwide doctors’ strike that lead to massive cancellations of routine diagnostic procedures.

There were no data collected on the impact of improved and timely diagnosis on patient management in terms of how soon patients could schedule surgical appointments, what procedures were performed, and identification of the short and midterm clinical outcomes. This was a significant limitation in this study, because adoption and funding of such a program more widely will require the demonstration of a favorable benefit-to-cost ratio.

Additional monitoring, training, and mentorship of MO, CO, interns, and technicians are needed on a regular basis to maintain the skills and interest in such a program. Establishing a cohort of MO well trained in FNAB and BMAT biopsy procedures, who can then train other MO and CO, would lead to increased numbers of those able to perform these procedures. Training in FNAB and BMAT biopsy procedures should be regarded as a basic requirement for all interns and MO, and these procedures should be recorded in logbooks as a requirement for continuing medical education. Training in FNAB and BMAT biopsy techniques should also be extended to residents in surgery, medicine, and radiology, so that as future consultants they will understand the roles and advantages of these procedures and be able to perform them. Furthermore, training in the use of increasingly inexpensive ultrasound imaging to guide FNABs is also needed, to increase the range of lesions that can be accessed by FNAB.

Rapid on-site evaluation^14^ should be a long-term goal for the FNAB clinics, to enhance patient care through immediate provisional reporting and triage of specimens and to reduce patient call-backs for insufficient material. This evaluation requires pathologists, or at least trainee pathologists, to be available to perform rapid on-site evaluation. To further reduce costs, FNAB should be available in all outpatient clinics, ideally on the patient’s first visit, to enhance patient care and avoid multiple return visits. In time, enhanced molecular and other ancillary testing of FNAB and BMAT biopsy materials can be incorporated to maximize the diagnostic usefulness of these simple tests.

We strongly recommend that a monitoring evaluation on framework be implemented at the sites of this program to encourage sustainability and to assess the impact of the program over a 3- to 5-year period. This would provide the data needed for appropriate recommendations for a wider adoption of this model.

This project has demonstrated that a model of task-shifting diagnostic procedures and skills from pathologists to MO and other medical personnel can be implemented successfully in a low-resource setting, and this can address some of the challenges associated with the shortage of pathologists. Furthermore, the project has shown that this can be accomplished by training pathology resident trainers centrally, using an experienced faculty, and then supporting these resident trainers to teach regional county hospital MO and CO. The project has decentralized and improved the FNAB and BMAT biopsy services in the county hospitals in line with government health policy.^15^ With additional government support and the collaboration of Kenyan and international pathologists, this project can potentially be rolled out across Kenya and similar LMIC settings.
